# New surgery approaches preserving entire papilla to treat isolated interdental intrabony defects: A narrative review

**DOI:** 10.1002/cre2.410

**Published:** 2021-02-16

**Authors:** Xiyan Pei

**Affiliations:** ^1^ First Clinic Division Peking University School and Hospital of Stomatology & National Clinical Research Center for Oral Diseases & National Engineering Laboratory for Digital and Material Technology of Stomatology & Beijing Key Laboratory of Digital Stomatology Beijing China

**Keywords:** entire papilla preservation, intrabony defects, modified vestibular incision subperiosteal tunnel access, nonincised papillae surgical approach, periodontal regeneration

## Abstract

**Objectives:**

To review novel techniques of preserving the entire papilla to minimize the trauma of fragile papilla in periodontal regeneration surgeries.

**Material and methods:**

Electronic databases (Pubmed) and relevant journals were searched until September 4, 2020. Randomized controlled trials, cross‐sectional and cohort studies in English were included. Three novel approaches of preserving the entire papilla were applied to bone regeneration for intrabony defects, which were entire papilla preservations (EPP), nonincised papillae surgical approach (NIPSA) and modified vestibular incision subperiosteal tunnel access (M‐VISTA).

**Results:**

Randomized control trials of the novel preserving entire papilla techniques were rarely reported. There were only case series or cohort studies. Several papilla preservation techniques (PPT) or minimally invasiveness surgical techniques (MIST) have existed for a long time. However, these techniques still have dissection at the papilla. There were no related RCTs comparing the three novel approaches of keeping the entire papilla with PPT and MIST. All three techniques showed totally primary wound closure. Compared with PPT/MIST, EPP and NIPSA seemed to have better clinical outcomes in reducing probing depth (PD) and clinical attachment level (CAL) gain.

**Conclusions:**

EPP and NIPSA seemed to have advanced results of PD reduction and CAL gain than PPT and MIST. This is needed to be confirmed by further research.

## 1 INTRODUCTION

Periodontal regeneration techniques are common treatments for intrabony defects. However, the techniques require high technique sensitivity and there are multiple factors related to the success. Surgery‐associated factors have an important impact on outcomes, apart from the patient‐related factors and defects‐related factors. Each step has an important role in the outcomes, such as incision design, flap pattern, debridement methods, materials position, flap reposition and suture. This review mainly focuses on the novel surgical approaches, rather than regeneration materials. Conventional periodontal flap surgery techniques used an incision to detach the interdental papilla (Cortellini & Tonetti, 2005, 2015). The incision at the interdental papilla may provide an excellent view of interdental defects. However, this also increased the risk of postoperative flap dehiscence and biomaterial exposure which are two main short‐term postoperative complications in early soft tissue healing stage. The lack of primary closure and membrane exposure may occur in 60% to 80% of the treated sites, especially when bone materials and membranes were used to fill the defects (Trombelli et al., 1997). Exposure of materials may be contaminated by bacteria and lead to failure of regeneration. During the secondary wound healing, shrinkage of the marginal soft tissues frequently occurred, which can cause gingival recession, tooth hypersensitivity and esthetic problems. The ideal design of incision and flaps should ensure primary closure of the flap and maintain space for regeneration at the interdental area.

To promote early soft tissue healing, minimize the trauma of papilla and reduce postoperative gingival recession, papilla preservation techniques (PPT) were proposed (Checchi et al., 2009; Cortellini et al., 1995; Di Tullio et al., 2013; Guarnieri, 2019; Miliauskaite et al., 2008). A series of minimally invasive surgical technique (MIST) were developed (Cortellini & Tonetti, 2007a, 2007b; Nibali et al., 2015, 2018). Papilla preservation flaps have evolved from traditional types to modified PPT (Checchi et al., 2009) and then to simplified PPT (Di Tullio et al., 2013). The incision of PPT was at the base of papilla. Although, these techniques could reduce papilla trauma to some extent compared with traditional techniques, the mesio‐distal dissection through papilla was still needed. Biomaterials beneath the incision line were still at risk of exposure. Followed the minimal invasiveness concept with the use of microscopes and microsurgical instruments, modified MIST has been developed (Barbato et al., 2020). Modified MIST usually raised the papilla only at the buccal or palatal side using a single flap approach, while MIST raised the papilla at both buccal and palatal sides with a double flap approach. It was also reported that the single flap approach and papilla preservation could provide better outcomes than the double flap (Barbato et al., 2020). Pushing the boundaries of minimal invasiveness, a minimally invasive non‐surgical (MINST) protocol has recently been proposed (Barbato et al., 2020; Nibali et al., 2015, 2018). Surgical microscopes and devices could provide better magnification, but these advanced techniques required a learning curve (Nibali et al., 2019).The above two series of techniques could reduce the rate of complication due to lack of closure and membrane exposure to 30% and 10% respectively (Cortellini & Tonetti, 2015). In periodontal surgery, minimal invasiveness is a trend for the treatment of intrabony defects. To reduce the complication rate and increase the outcome of PPT and MIST, several novel surgical procedures have been developed lately to preserve the entire papilla without dissection. Maintaining papilla integrity and soft tissue profile are the keys to reducing the complications, particularly in the esthetic area.

In the study, we searched electronic databases (Pubmed) and relevant journals until September 4, 2020 including all kinds of papers as randomized controlled trials, cross‐sectional and cohort studies in English. Several PPT or minimally invasiveness surgical techniques (MIST) have existed for a long time. However, these techniques still have dissection at the papilla. Three novel approaches of preserving the entire papilla were applied to bone regeneration for intrabony defects, which were entire papilla preservations (EPP), nonincised papillae surgical approach (NIPSA) and modified vestibular incision subperiosteal tunnel access (M‐VISTA). There were no related RCTs comparing the three novel approaches of keeping the entire papilla with PPT and MIST. This study aims to review novel techniques of preserving the entire papilla to minimize the trauma of fragile papilla in periodontal regeneration surgeries.

## 2 REVIEW

### 2.1 Entire papilla preservation technique

**FIGURE 1:**
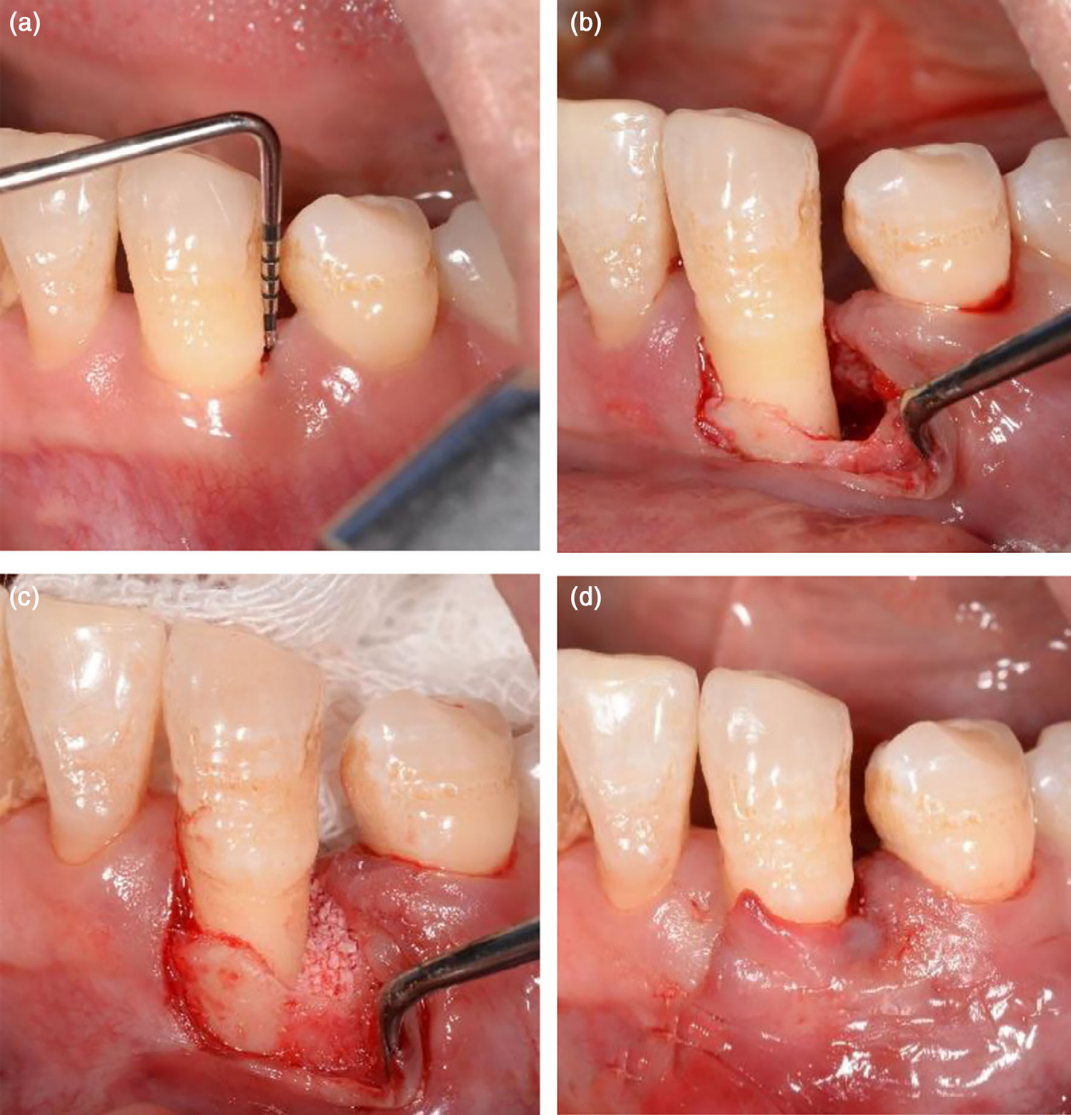
The entire papilla preservation (EPP) technique. (a) Pre‐operation; (b) vertical incision at neighboring papilla and preserve the intact papilla above the intrabony defects; (c) debridement and placement of biomaterials; (d) 6–0 absorbable monofilament suture by interrupted suturing at the vertical incision

EPP technique was proposed in 2017 (Aslan et al., 2017a, 2017b) to preserve the whole integrity of the defect‐associated papilla providing a tunnel‐like undermining incision. The completely preserved papilla provided an intact gingival chamber to stabilize the blood clot and improved the wound healing. EPP required a short buccal vertical releasing incision of the nearby tooth extending just beyond the mucogingival line. Following the elevation of a buccal full‐thickness flap extending from the vertical incision to the defect‐associated papilla, an angled tunneling instrument was used to prepare the undermining tunnel of the papilla. The papilla was also elevated in a full‐thickness manner. Wound healing in vertical incision line was reported without any complications. Besides, the papilla was fully nourished through its native uninterrupted vascular supply, thus wound exposure could be avoided. It was also reported (Aslan et al., 2020) that the early healing phrase was uneventful in all cases and 100% of primary wound closure was maintained in 1 year. EPP technique without combination of any biomaterials showed the outcomes in terms of CAL gain (5.83 ± 1.12 mm), PD reduction (6.2 ± 1.33 mm) and gingival recession (0.36 ± 0.54 mm). Application of EPP with or without regenerative biomaterials resulted in significant outcomes of CAL gain and PD reduction, with negligible increase in gingival recession. A case was shown in Figure 1. There was a deep pocket at the distal site of left mandibular canine. By using the EPP technique with a small vertical incision, the defect was exposed appropriately and minimally. The papilla at the defect site was totally preserved.

### 2.2 Nonincised papillae surgical approach

**FIGURE 2:**
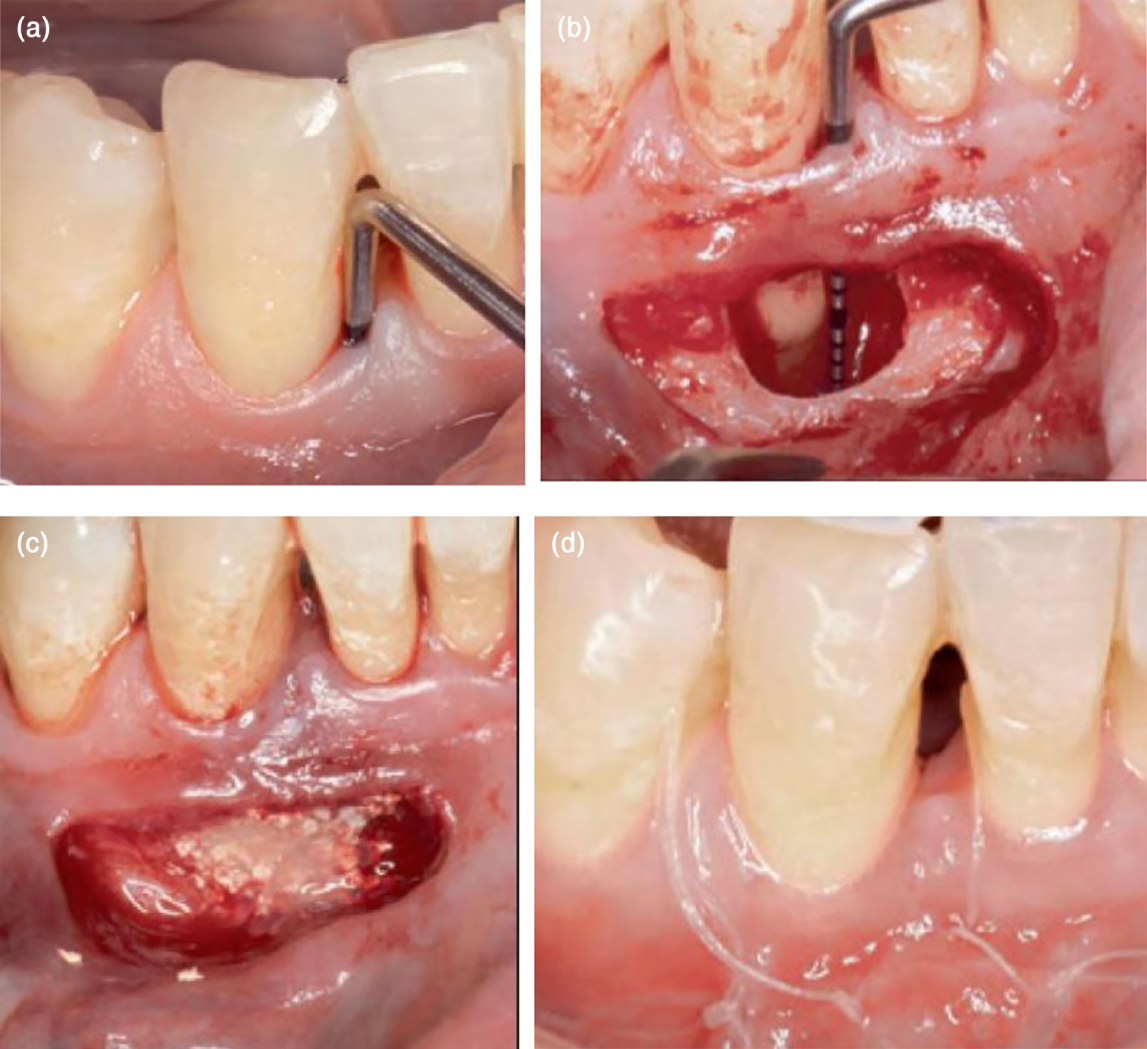
Nonincised papillae surgical approach (NIPSA) (Moreno Rodriguez & Caffesse, 2018). (a) Pre‐operation; (b) horizontal incision as apical as possible and preserve the intact papilla above the intrabony defects; (c) debridement and placement of biomaterials; (d) absorbable monofilament suture by interrupted suturing at the horizontal incision

NIPSA was proposed in 2018 (Moreno Rodriguez & Caffesse, 2018). The basic principle of the technique was the placement of only buccal horizontal incision at the mucosa, as apically as possible from the periodontal defects and the marginal tissues. The raising of a mucoperiosteal flap coronally permitted apical access to the defect while leaving the marginal tissues intact. Meanwhile, the lingual soft tissues were also intact. The marginal soft tissues acted as a roof to protect the underlying interproximal defects and prevented collapse of papilla. It should be kept in mind that the mapping of the intrabony defects and place the horizontal incision correctly which should be always situated on the cortical bone. The incision was extended mesiodistally to expose the cortical bone around the defects. The technique could offer several clinical advantages. The flap could be easily stabilized on the attached marginal tissues to achieve wound closure by preserving blood clots. By leaving a great volume of intact supracrestal soft tissue, better preservation of the blood supply in the interdental area eventually achieved. Postsurgical shrinkage was minimized by this technique. Although the supraperiosteal gingival vessels near the mucogingival junction were dissected, the nonincised gingival vessels showed continuity with periodontal ligament and lingual blood supply was rich. It had advantages over traditional extended flap in terms of blood supply. However, the blood supply was no better than the other two EPP techniques because the horizontal incision damaged the apical blood supply. During debridement, the 2‐3 mm of marginal tissues were kept unaltered. The report (Moreno Rodriguez, Ortiz Ruiz, & Caffesse, 2019) revealed that PD reduction was 5.53 ± 2.56 mm, CAL improvement was 5.33 ± 2.47 mm, recession increase was 0.20 ± 0.41 and early wound healing index at 1 week was 1.5 ± 0.7. The mucogingival line remained unchanged. NIPSA resulted in significant CAL gain and PD reduction, and meaningfully NIPSA showed a lower postoperative gingival recession of the interdental papilla and more CAL gain than MIST (Moreno Rodriguez, Ortiz Ruiz, & Caffesse, 2019). Early wound closure in NIPSA was better than MIST. Complete wound closure was present in 11 out of 15 cases in NIPSA group and 6 out of 15 cases in the MIST group. There were no incomplete wound closure cases in NIPSA, while 5 cases in MIST group (Moreno Rodriguez, Ortiz Ruiz, & Caffesse, 2019). A case was shown in Figure 2. There was a deep pocket at the mesial site of right mandibular canine. Using the NIPSA, the defect was exposed without incising the marginal soft tissues and the papilla.

### 2.3 Modified vestibular incision subperiosteal tunnel access

**FIGURE 3:**
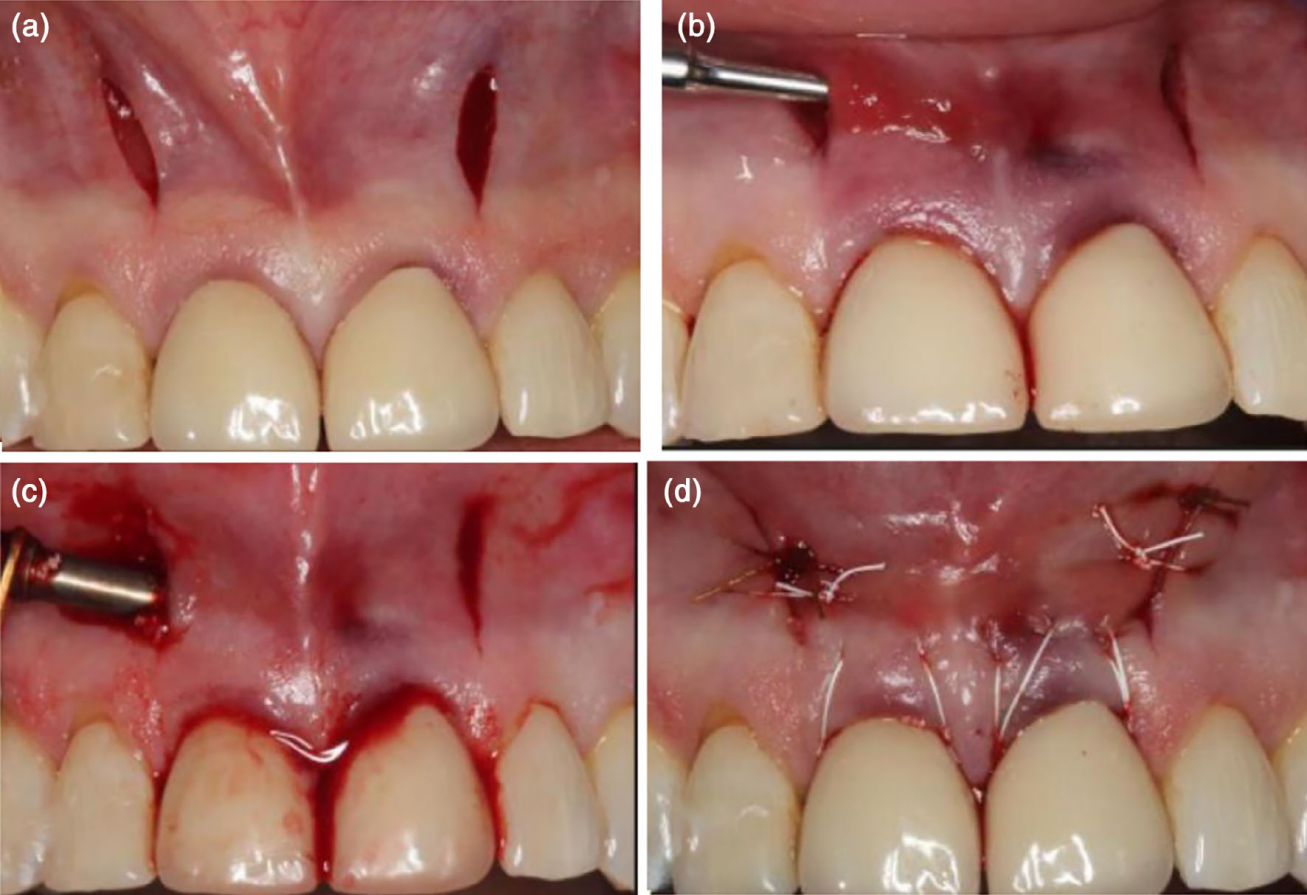
Modified vestibular incision subperiosteal tunnel access (M‐VISTA) (Najafi et al., 2018). (a) Pre‐operation; (b) vertical incision at vestibular position and preserve the intact papilla above the intrabony defects; (c) debridement and placement of biomaterials; (d) absorbable monofilament suture by interrupted suturing

M‐VISTA (Najafi et al., 2018) was applied for treating intrabony defects in the esthetic area. In the past, subperiosteal tunnel access has been used for ridge augmentation through small vestibular incisions and minimal tissue dissection to access the site without jeopardizing the soft tissue profile. In addition to ridge augmentation in implant surgery, the technique was widely used for root coverage through either supraperiosteal or subperiosteal tunnel way (Schulze‐Spate & Lee, 2019). Using a tunneling approach may prevent some potential complications of raising a flap. The vertical incision located near the intrabony defects providing adequate access to the defects. This vertical incision was made beyond the mucogingival line. Intrasulcular incisions were made on midfacial surfaces from one line angle to the other of each tooth, avoiding the papillae. Subperiosteal tunnel elevation was performed using elevators. Unlike VISTA technique using partial‐thickness flap above the periosteum for root coverage, VISTA usually raised the periosteum to form a full‐thickness flap to place the bone materials for treating intrabony defects. Coronally anchored suturing technique bonded on facial surfaces of the teeth was applied to advance the mucogingival complex coronally. Because of this, a minimum 2 mm keratinized gingival width might be required to maintain gingival health. Only a case series revealed successful outcomes of no postoperative gingival recession in clinical and radiographic follow‐up of 18 months. A case using the M‐VISTA was shown in Figure 3. The incision was located at the vestibular area and the flap was raised in a tunnel way.

## 3 DISCUSSION

Compared with the conventional papilla preservation and minimally invasive techniques, EPP and NIPSA seemed to have better clinical results (Table 1). PD reduction ranged from 5.5 to 6.5 mm, CAL gain ranged from 5.3 to 6.3 mm and GR was 0.2 mm to 0.36 mm in EPP and NIPSA. Reviewing the literatures on conventional EPP, a long‐term papilla preservation flap surgery in esthetic area showed the outcomes that PD reduced by 4.2 mm, CAL gained by 4.05 mm, no obvious recession increase occurred (Guarnieri, 2019). It was reported that MIST could show PD reduction of 4.24 mm, CAL gain of 3.89 mm and GR increase of 0.44 mm, bone fill gain of 58.25% and VAS value of 1.16 (Clementini et al., 2019). There were no related results for M‐VISTA. There were only case reports or cohort studies on EPP and NISPA. There were some similarities of the three techniques:

1. The indications were limited to some particular types of intrabony defects. A 2‐wall intrabony with a missing buccal bony wall and a relatively well‐preserved lingual wall was the best indication. In other words, if the defects were involved lingual bone crest, the three approaches would not be appropriate.
2. The interdental papilla was totally preserved without being dissected as to maitain the integrity of papilla.
3. The three approaches used full thickness flap to access the defects, which was similar to the conventional bone regeneration periodontal surgeries. This was unlike the mucogingival VISTA surgeries for root coverage in which partial thickness flap was used.
4. Bone grafts were usually applied, while membranes were not necessary, especially non‐ absorbable membranes. Soft tissue grafts like CTG could be added if indicated.

The indifferences were as below:

1. M‐VISTA was recommended for the anterior area, while EPP was indicated not for using at anterior zone as there might be a scar left at the vertical incision line.
2. NIPSA kept the marginal gingival tissues unaltered, while the other two raised the gingival soft tissues. EPP was an open way and M‐VISTA acted in a tunnel manner.
3. The blood supply of NIPSA was no better than that of EPP and M‐VISTA, as the horizontal incision cut off some blood supply to the interdental papilla.
4. M‐VISTA was no longer suitable when keratinized tissue width was less than 2 mm, as the flap would be repositioned coronally. While the other two techniques did not have the limitation.
5. The suture of EPP was the simplest and only interrupted sutures were needed, while the other two should use special advanced techniques like horizontal mattress and sling sutures.

**TABLE 1:** Comparison of three entire papilla preservation (EPP) techniques

	EPP	NIPSA	M‐VISTA
Incision position	Vertical releasing incision at neighboring papilla	Horizontal incision as apically as possible at the apical of the defect	Two small vertical vestibular incisions at adjacent teeth
Details about incision	The vertical releasing incision was short and does not exceed the mucogingival line	The incision was as apically as possible. The incision was always situated on the cortical bone.	(1) Labial frenulum was not an optimal location for incision as stability was critical for regeneration (2) The incision located beyond the mucogingival line
Flap pattern	Open flap; Tunnel of the defect‐associated papilla; The marginal soft tissues were raised.	Open flap; The marginal 2‐3 mm soft tissue must be kept unaltered.	Tunnel way; The marginal soft tissue was accessed in a tunneling way.
Relation with periosteum	Subperiosteum/full thickness flap	Subperiosteum/full thickness flap	Subperiosteum/full thickness flap
Indications	Interdental isolated intrabony defects without involving lingual sites. Anterior and posterior area	Interdental isolated intrabony defects without involving lingual sites. Anterior and posterior area	Interdental isolated intrabony defects without involving lingual sites. Anterior esthetic area
Bone or soft grafts	Enamel matrix derivative (EMD). Bovine‐derived bone substitutes (BS). No bone or soft tissue graft	Enamel matrix derivative (EMD). HA‐bovine bone xenograft. No soft tissue graft/CTG	Enamel matrix derivative (EMD). Demineralized free‐dried bone allograft (DFDBA). No soft tissue graft
Flap reposition	Reposition originally	Reposition originally	Coronally ≥2 mm above the CEJ
suture	Interrupted sutures	Horizontal mattress sutures; Interrupted sutures; A double‐sling suture	Modified horizontal mattress anchoring technique and interrupted sutures
Outcome	PD reduction: 6.5 ± 2.65 mm (EMD + BS) 6.2 ± 1.33 mm (none) CAL gain: 6.3 ± 2.5 mm (EMD + BS) 5.83 ± 1.12 (none) GR increase: 0.2 ± 0.25 (EMD + BS) 0.36 ± 0.54 (none) (Aslan et al., 2020) 100% early wound closure	PD reduction: 5.53 ± 2.56 mm CAL gain: 5.33 ± 2.47 mm GR increase: 0.20 ± 0.41 mm early wound healing index at 1 week: 1.5 ± 0.7 (Moreno Rodriguez, Ortiz Ruiz, & Caffesse, 2019)	None
Limitations	Scar at the vertical incision A narrow interdental space with a high risk of tearing the fragile papilla was not suggested	Blood supply was interrupted by horizontal incision	Keratinized tissue width ≥ 2 mm

Abbreviations: CEJ, cementoenamel junction; CTG, connective tissue graft; EPP, entire papilla preservation; M‐VISTA, modified vestibular incision subperiosteal tunnel access; NIPSA, nonincised papillae surgical approach.

Thus, the three novel techniques should be applied according to the specific indications. In the future, more large‐scale studies should be conducted to compare the three novel techniques. Meanwhile, more convincing evidence like RCTs should be conducted to confirm if the novel techniques were superior to EPP and MIST.

## 4 CONCLUSIONS

This review introduced three novel techniques for preserving the entire papilla to treat intrabony defects. The techniques went further to preserve the intact papilla than traditional PPT and MIST to reduce trauma. EPP/NIPSA seemed to have better clinical results of PD reduction and CAL gain than PPT/MIST. However, it was still needed to be confirmed by further research. The outcomes of M‐VISTA should be reported in the future.

## CONFLICT OF INTEREST

The author states that there are no conflict of interest.

## AUTHOR CONTRIBUTIONS

Xiyan Pei contributed all the work of design, review and writing work.

## ETHICS STATEMENT

The review is declared with the Helsinki Declaration of 1975, as revised in 2013.

## Data Availability

Data openly available in a public repository that issues datasets with DOIs.
